# Increased Vasoactive Intestinal Peptide (VIP) in polycystic ovary syndrome patients undergoing IVF

**DOI:** 10.3389/fendo.2024.1331282

**Published:** 2024-05-07

**Authors:** Luana Sallicandro, Elko Gliozheni, Davide Feudi, Paola Sabbatini, Roberto Maria Pellegrino, Husam B. R. Alabed, Domenico Baldini, Sandro Gerli, Carlo Alviggi, Eliano Cascardi, Ettore Cicinelli, Antonio Malvasi, Bernard Fioretti

**Affiliations:** ^1^ Department of Chemistry, Biology and Biotechnologies, University of Perugia, Perugia, Italy; ^2^ Department of Medicine and Surgery, Perugia Medical School, University of Perugia, Perugia, Italy; ^3^ In Vitro Fertilization (IVF) Center, Momo Fertilife, Bisceglie, Italy; ^4^ Department of Obstetrics and Gynecology, Centre of Perinatal and Reproductive Medicine, University of Perugia, Perugia, Italy; ^5^ Department of Clinical Gynecological Emergency, Obstetrics and Reproductive Medicine, University Federico II, Naples, Italy; ^6^ Department of Medical Sciences, University of Turin, Turin, Italy; ^7^ Department of Interdisciplinary Medicine (DIM), University of Bari “Aldo Moro”, Bari, Italy

**Keywords:** PCOS (polycystic ovarian syndrome), VIP (vasoactive intestinal peptide), noradrenalin (NA), follicular fluid, In Vitro Fertilization (IVF), metabolomic, ovarian innervation

## Abstract

**Introduction:**

Polycystic ovary syndrome (PCOS) is a common multifactorial and polygenic disorder of the endocrine system, affecting up to 20% of women in reproductive age with a still unknown etiology. Follicular fluid (FF) represents an environment for the normal development of follicles rich in metabolites, hormones and neurotransmitters, but in some instances of PCOS the composition can be different. Vasoactive intestinal peptide (VIP) is an endogenous autonomic neuropeptide involved in follicular atresia, granulosa cell physiology and steroidogenesis.

**Methods:**

ELISA assays were performed to measure VIP and estradiol levels in human follicular fluids, while AMH, FSH, LH, estradiol and progesterone in the plasma were quantified by chemiluminescence. UHPLC/QTOF was used to perform the untargeted metabolomic analysis.

**Results:**

Our ELISA and metabolomic results show: i) an increased concentration of VIP in follicular fluid of PCOS patients (n=9) of about 30% with respect to control group (n=10) (132 ± 28 pg/ml versus 103 ± 26 pg/ml, p=0,03) in women undergoing *in vitro* fertilization (IVF), ii) a linear positive correlation (p=0.05, r=0.45) between VIP concentration and serum Anti-Müllerian Hormone (AMH) concentration and iii) a linear negative correlation between VIP and noradrenaline metabolism. No correlation between VIP and estradiol (E2) concentration in follicular fluid was found. A negative correlation was found between VIP and noradrenaline metabolite 3,4-dihydroxyphenylglycolaldehyde (DOPGAL) in follicular fluids.

**Conclusion:**

VIP concentration in follicular fluids was increased in PCOS patients and a correlation was found with noradrenaline metabolism indicating a possible dysregulation of the sympathetic reflex in the ovarian follicles. The functional role of VIP as noradrenergic modulator in ovarian physiology and PCOS pathophysiology was discussed.

## Introduction

Polycystic ovary syndrome (PCOS) is an endocrinopathy with a prevalence in 5-20% in women of reproductive age (1–3). During the last decade there has been much discussion about the pathogenesis of PCOS, a multifactorial disorder that shows as common features oligo/anovulation, polycystic aspect of the ovaries on ultrasound and hyperandrogenism (4). It is known that environmental and genetic factors seem to be implicated (5, 6). These factors may contribute to infertility, increased risk of endometrial carcinoma and cardiovascular disease (7).

Some of these aspects are associated with chronic sympathetic nervous system hyperactivity, thus suggesting the hypothesis that PCOS pathogenesis could be under the influence of the nervous system (8). It still remains unclear whether this occurs as a consequence of the condition or if it plays a pathological role in PCOS development (9).

The autonomic nervous innervation of the ovary is complex and it includes sympathetic (10) and parasympathetic efferent fibers (11), as well as visceral afferent (sensory) fibers (12), connected through multisynaptic pathways to the central nervous system (12, 13). The sympathetic fibers connected with the ovary reach most of its components, such as blood vessels, interstitial tissue and follicular wall, but do not intercept granulosa cells and the corpus luteum (14). In the mammalian model, the ovary is innervated by two sympathetic sources, namely the ovarian plexus nerve and the suspensory ovarian nerve (15). The latter, arising from the superior mesenteric celiac ganglion (CSMG), innervates interstitial and theca cells. In addition, there is evidence from several studies that catecholamines and their metabolites may be involved in a neuronal communication circuit with the ovary, regulating its function; this would be through CSMG which is the main source of Vasoactive Intestinal Peptide (VIP) and noradrenaline (NA) (16–18). Histochemical studies of catecholamine fluorescence in the human ovary have identified a network of fibers penetrating the ovary through the hilar region and distributed through the perivascular region, the stroma and the follicular theca layers (19, 20). In the ovary of the rat, it was demonstrated that the sympathetic nerves are able to influence ovarian secretory activity since NA and VIP potentially stimulate steroid secretion (10).

VIP is a highly basic linear 28 amino-acid peptide (21, 22), firstly isolated from porcine duodenum (23). It exhibits a plethora of biological activities, ranging from relaxation of tracheobronchial and gastrointestinal smooth muscle to the support in endocrine and intestinal chloride secretion (22, 24–26). Both VIP and VIP-ergic nerve fibers have been found in the ovaries of animal models and humans, mainly close to the smooth muscle of blood vessels and also within the ovarian stroma (27, 28).

This neuropeptide regulates several ovarian functions such as follicular development, ovulation, steroidogenesis and granulosa cell apoptosis (29). VIP is recognized by two receptors that are VPAC1-R and VPAC2-R, which belong to the class B of G-protein-coupled receptors (GPCRs) (30). VPAC1-R is predominantly found at the level of the ovarian hilus, where the ovarian arteries enter the ovary. Indeed, VPAC1-R is correlated to blood vessel walls but it is also expressed in the stroma near follicles as well as in theca and granulosa cells, while VPAC2-R is expressed only in granulosa cells (31, 32). Given the clinical and anatomical features, a possible role of VIP is expected in PCOS, since the density of sympathetic nerve fibers is increased in polycystic ovaries of women (33), rats (34), and pigs (35, 36). Several neuropeptidergic fibers result to be overexpressed in PCOS women, including VIP-ergic ones, supporting our interest in this topic (9).

To investigate the function of VIP in PCOS in PCOS, we decided to study the human follicular fluid (FF) because its composition depends on local follicular metabolic processes and on the biological activities of ovarian cells (37). In addition biological material, this is easily available as it is a waste material of the *in vitro* fertilization (IVF) technique. Having a more comprehensive view of the follicular fluid could be considered as an excellent resource for a better understanding PCOS. To this end, the aim of the present study was to compare the concentration of VIP among PCOS and non-PCOS follicular fluid of women that underwent IVF treatment. After this analysis, we deepen our investigation of the physiopathological consequences of putative VIP dysregulation, including its possible correlation with the hormones (estradiol, in particular). Furthermore, an untargeted metabolomic profiling of the FF samples has been performed, to detect the possible modifications of noradrenaline and its metabolites in PCOS.

## Materials and methods

### Patients

Nineteen women undergoing IVF treatment were enrolled at the Center of Reproductive Medicine and IVF Unit in Perugia (Perugia, Italy), between July 2023 and October 2023. All patients signed informed consent. The study protocol was approved by the local Ethical Committee (no. 27139) and recorded on ClinicalTrials.gov with ID: NCT05958914. In all patients BMI, basal FSH, number of antral follicles were evaluated. Women with PCOS were diagnosed according to the Rotterdam criteria (38). Ovarian stimulation was performed in accordance to the ovarian response obtained, serial ultrasound examinations and serum routine serum hormonal assessments conducted every two days every two days (follicular stimulating hormone, FSH; luteinizing hormone, LH; estradiol, E2, progesterone, P4); during the stimulation the dose of gonadotropins was adjusted as needed. Patients with mutated FSH receptors were excluded from the study. During the recruitment, 19 patients were enrolled and the clinical information of all participants is presented in **Table 1**. AMH levels were measured by chemiluminescence quantitative determination CLIA using MAGLUMI 800, while FSH, LH, estradiol and progesterone levels were determined by quantitative chemiluminescence using CL 1200 I Mindray.

**Table 1:** Clinical and endocrine parameters of patients undergoing IVF during assisted reproductive technique.

Features *Clinical characteristic*	CTRL mean ± sd	PCOS mean ± sd	P value
**Age, years**	31.5 ± 2.9 (n=9)	35.3 ± 4.1 (n=9)	0.09
**Height, m**	1.64 ± 0.03 (n=10)	1.61 ± 0.04 (n=9)	0.14
**Weight, kg**	67.8 ± 13.8 (n=10)	63.5 ± 5.0 (n=9)	0.53
**BMI, kg/m^2^**	24.9 ± 4.4 (n=10)	24.1 ± 2.1 (n=9)	0.83
**FSH, mUI/ml**	7.3 ± 1.9 (n=10)	5.8 ± 0.8 (n=9)	0.14
**LH, mUI/ml**	6.7 ± 2.8 (n=10)	6.2 ± 3.1 (n=9)	0.79
**17βestradiol, pg/ml**	55.7 ± 26.4 (n=9)	37.9 ± 23.3 (n=9)	0.3
**AMH, ng/mL**	1.6 ± 0.6 (n=10)	6.47 ± 2.8 (n=9)	0.003
**Progesterone, ng/ml**	0.7 ± 0.3 (n=7)	0.5 ± 0.11 (n=9)	0.23
**rFSH starting, UI**	255 ± 38 (n=10)	216 ± 17 (n=9)	0.001
**rFSH cumulative, UI**	2906 ± 522 (n=10)	2345 ± 311 (n=9)	0.001
**17βestradiol trigger, pg/ml**	1238 ± 669 (n=9)	3064 ± 1138 (n =9)	0.0008
**Progesterone trigger, ng/ml**	0.93 ± 0.65 (n=7)	1.07 ± 0.6 (n =9)	0.68

The clinical characteristics and endocrine parameters are presented as mean ± standard deviation (sd). Statistical analysis was performed by using t test between PCOS and control group. AMH, anti-Mullerian hormone; BMI, body mass index; FSH, follicle- stimulating hormone; LH, luteinizing hormone; PCOS, polycystic ovary syndrome.

### Collection of follicular fluid samples

FF was obtained after oocyte retrieval during IVF according to the following procedure. The follicular fluid aspirated from each patient was pooled in conical bottomed 15 ml polypropylene tubes and centrifugated at 1000 g for 3 minutes at 21°C. The supernatant was collected, transported to the laboratory and stored at -80°C until further analysis.

### VIP enzyme-linked immunoassay

The enzyme-linked immunosorbent assay (ELISA) used to quantify VIP in follicular samples was based on the principle of sandwich enzyme immunoassay. The quantification was performed following manufacturer’s protocol of ELISA kit (ELK Biotechnology CO., Ltd). Briefly, the microtiter plate provided has been pre-coated with an antibody specific for VIP. 100 μL of standards and samples were added to the appropriate microtiter plate wells and incubated for 80 minutes at 37°C. After the wash, 100 μl/well of biotin-conjugated antibody solution was added and incubated for 50 minutes at 37°C. After further washes, 100 μl of avidin conjugated to horseradish peroxidase (HRP) solution was added to each microplate well and incubated for further 50 minutes at 37°C. Next, a final wash was performed and 90 μL of 5-5’-tetramethylbenzidine (TMB) substrate solution was added and the color development was monitored and quantified after the addition of sulfuric acid solution. The absorbance was measured spectrophotometrically at 450 nm by using microplate spectrophotometer (Labsystem Multiskan MS). Standard curve was obtained in the same way of sample treatment by using VIP standard solutions according to manufacturer’s instructions. The concentration of VIP in the samples was determined by comparing the optical density of the samples to the standard curve.

### Estradiol enzyme-linked immunoassay

ELISA was used to quantify E2 in follicular samples based on competitive inhibition enzyme immunoassay technique. The quantification was performed following the manufacturer’s protocol of ELISA kit (ELK Biotechnology CO., Ltd). The microtiter plate provided was pre-coated with E2 protein. Standards and samples were added with a biotin-conjugated antibody specific to E2. Afterward, HRP was added and incubated. After a final wash, TMB substrate solution was added, incubated for a brief time, and then, the reaction was stopped with the addition of sulphuric acid solution. The color change was measured spectrophotometrically at a wavelength of 450 nm with the same method described in the section regarding VIP enzyme-linked immunoassay.

### Untargeted ultra-performance liquid chromatography mass spectrometry (UHPLC)-QTOF-based metabolomic and statistical analysis

Extraction of metabolites from Follicular Fluid: A total of 50 μl of freshly thawed follicular fluid was combined with 50 μl of H2O and 400 μl of 100% methanol in a 1.5 ml Eppendorf tube. Following vigorous shaking for 10 seconds, the tubes were placed at -80°C for 30 minutes. Subsequently, the tubes underwent centrifugation at 16,000 rcf for 20 minutes at 4°C. 400 μl of the resulting supernatant were transferred to an autosampler glass vial and subjected to drying using a gentle flow of nitrogen. The dried residue was reconstituted with 40 μl of a 2:8 water/acetonitrile mixture and promptly subjected to UHPLC/MS-QTOF analysis.

Untargeted mass analysis: UHPLC/MS-QTOF was performed using a 1260 Infinity II LC System coupled with an Agilent 6530 Q-TOF spectrometer (Agilent Technologies, Santa Clara, CA USA). The LC consists of a quaternary pump, a thermostated column compartment, and an autosampler. Separation was carried out on a WATERS X-Bridge BEH amide, 2.1x150 mm, 2.5 μm column at 45°C, and 0.35 mL/min flow. The mobile phase consisted of water (A) and water/acetonitrile 15:85 (B) both containing 0.2% of formic acid. The gradient was: time 0–3 min isocratic at A 10%, B 90%; time from 3 to 13 min: linear-gradient to A 52%, B 48%; time 13–15 min isocratic A 52%, B 48%; time from 15 to 16 min: linear-gradient to A 10%, B 90%; time 20 min: stop run. Spectrometric data was acquired in the 40–1700 m/z range both in negative and positive polarity. The Agilent JetStream source operated as follows: Gas Temp (N2) 280 C, Drying Gas 12 L/min, Nebulizer 50 psi, Sheath Gas temp: 300°C at 12 l/min. Raw data was processed using MS-DIAL software (4.48) to perform peak-picking, alignment, peak integration and data annotation. The MS signal threshold was set at 1000 counts. The annotation of the metabolites was conducted on the basis of MS and MS/MS signal using the NIST2020 tandem database. Finally, a data matrix was obtained reporting the metabolite name and area of each peak revealed in each sample analyzed. To conduct the statistical analysis and pathway analysis, the relative abundances of the metabolites (in terms of areas under the peaks) were normalized by median. In addition, a logarithmic transformation of the data was performed, followed by Pareto scaling. Subsequently, this data was compared and studied using the MetaboAnalyst platform.

### Targeted HPLC-MS analysis

Human follicular fluids were treated with cold ethanol (2 times the volume) at -20°C for 60 minutes and then centrifugate at 15000 g for 5 min to eliminate the protein fraction. The supernatant was then submitted to analysis with a JASCO HPLC coupled with a Single Quadrupole Mass Spectrometer (Advion ExpressionL) mode using an Agilent Poroshell 120 C18 column (4.6 mm x 150 mm, 2.7 µm). The mobile phase A consisted of water with 0.1 formic acid and acetonitrile (90:10, v/v), the mobile phase B consisted of water with 0.1 formic acid and acetonitrile (50:50, v/v). The HPLC gradient elution, at a flow rate of 0.5 mL/min, was 10% B at 0-1 min, 10%-100 B at 1-9 min, 100% B at 9-10 min. When sequences of analysis were performed, the gradient was 100%-10% B at 10-11 min and 10% B at 11-13 min. The MS analysis was performed in an ESI+ Single Ion Monitoring (reference mass: 168.2 ± 0.2) and in scan mode (scanning from 100 to 800).

### Statistical analysis

Fisher’s chi square, Pearson correlation coefficient and the T-student test were used to analyze data, since the they were normally distributed, with R statistical Software (v.3.0; R Core Team (2023)) through R Studio ((Posit team 2023). R Studio: integrated Development Environment R. Posit Software, PBC, Boston, MA) the ggpubr package was used to perform graphical analyses (39). Each result is reported with relative standard deviation and relative statistical significance (p<0.05) obtained with the Student’s t test. Scatter plots were generated with Origin 6.1.

## Results

### VIP concentration was found to be increased in the follicular fluid of PCOS patients

For this study 19 patients were enrolled, 9 PCOS and 10 non-PCOS women according to Rotterdam criteria (see Methods). Clinical and endocrine parameters of controls (non- PCOS) and PCOS patients are displayed in **Table 1**. There was no significant anthropometric differences in mean ages, height, weight and BMI between PCOS patients and controls. In contrast, the serum anti-Müllerian (AMH) hormone level was significantly higher in PCOS group (P<0.05), in accordance to previous diagnostic studies (40). The concentration of LH, basal E2 and basal P4 didn’t differ between the two groups, while a trend to lower FSH levels was found in the PCOS group compared to control (p<0.5). The stimulation protocol was different between the two groups since the rFSH starting dose and rFSH cumulative dose was lower in PCOS with respect to the non-PCOS, according to an increased ovarian sensibility to gonadotropins (41) (**Table 1**).

**Figure 1:**
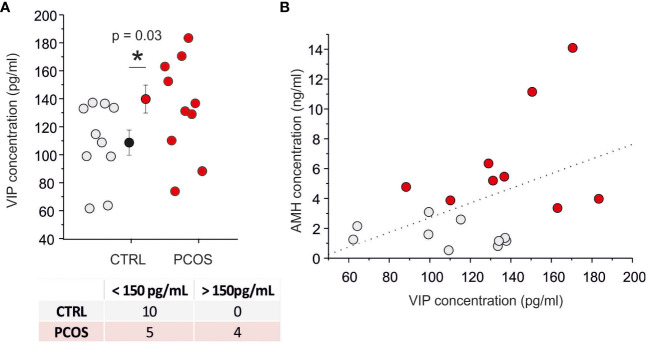
VIP levels are increased in the PCOS group. **(A)** Scatter plot of VIP concentration on follicular fluid in control (gray) and PCOS (red) patients. The contingency table represented in the bottom divide the PCOS and CTRL samples into two populations by using a threshold of 150 pg/ml (Fisher’s exact test=0.03). **(B)** Relationship between anti-Müllerian hormone (AMH) serum level and VIP level in follicular fluid. The dash line represents the best linear fit (p=0.053) with a correlation index of 0.45. (*) indicates the significance with p< 0,05.

By using ELISA assay, we measured the VIP concentration in FF recovered during oocyte pick-up. The FF-VIP concentration was significantly higher in women with PCOS compared to controls (p<0.05; FF-VIP in the PCOS population ranged from 88 pg/ml to 183 pg/ml with a mean of 132 ± 28 pg/ml (n = 9) whereas FF-VIP in non PCOS ranged from 62 pg/ml to 138 pg/ml with a mean of 103 ± 26 pg/ml (n=10); **Figure 1A**). A weak linear correlation between serum AMH level and follicular fluid VIP was found (r=0,45; p=0.05; **Figure 1B**) whereas no correlation was observed between age, BMI, E2, FSH, LH and P4 levels (data not shown). A specific subpopulation characterized by VIP concentration (>150 pg/ml) was statistically observed only in PCOS group compared with controls as demonstrated by Fischer’s test (p=0.03) (bottom table of **Figure 1A**). All these data indicate the presence of neuronal dysregulation of VIP-ergic neurotransmission.

In addition, as an internal control, we investigated the biological stability of VIP in follicular fluid. A significant reduction of VIP concentration was observed in time (see **Supplementary Figure 1** for details) indicating the presence of a degradation pathway for this neuropeptide.

### VIP level correlates with NA turnover

**Figure 2:**
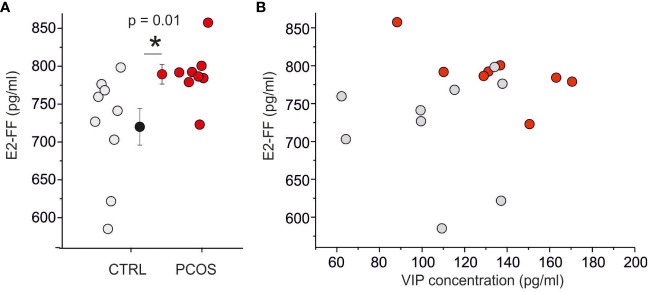
E2 levels are are not correlated with VIP concentration. **(A)** Scatter plot of E2 concentration on follicular fluid in control (gray) and PCOS (red) patients. **(B)** Relationship between E2 and VIP levels in follicular fluid. To be noted the absence of correlation between VIP and E2. (*) indicates the significance with p< 0,05.

FF is surrounded by granulosa cells, where VIP receptor activation stimulates estrogen and progesterone production (10, 28); based on this consideration, we decided to investigate the correlation between VIP concentration and steroidogenesis. Considering that at the time of the trigger plamsmatic concentration of E2 was significatively increased in PCOS patients with respect to non-PCOS women (p<0.005; E2 trigger in PCOS is 3064 ± 1138 ng/ml, while E2 trigger in non-PCOS is 1238 ± 669 ng/ml, **Table 1**), while no difference was observed in P4 levels (p>0.05; P4 trigger in PCOS is 1.07 ± 0.6 ng/ml, while P4 trigger in non-PCOS is 0.93 ± 0.65 ng/ml, **Table 1**), we decided to perform an ELISA to quantify the E2 in the same FF used for VIP assessment. The E2 concentration in FF was significantly higher in women with PCOS compared with controls (p < 0.05; E2-FF in the PCOS population ranged from 723 pg/ml to 857 pg/ml with a mean of 789 ± 13 pg/ml (n = 8) whereas E2-FF in non PCOS ranged from 585 pg/ml to 798 pg/ml with a mean of 720 ± 24 pg/ml (n=9); **Figure 2A**). A good correlation (R = 0.44) between plasma and follicular E2 concentration was found, while no correlation was observed between E2-FF and VIP (**Figure 2B**).

**Figure 3:**
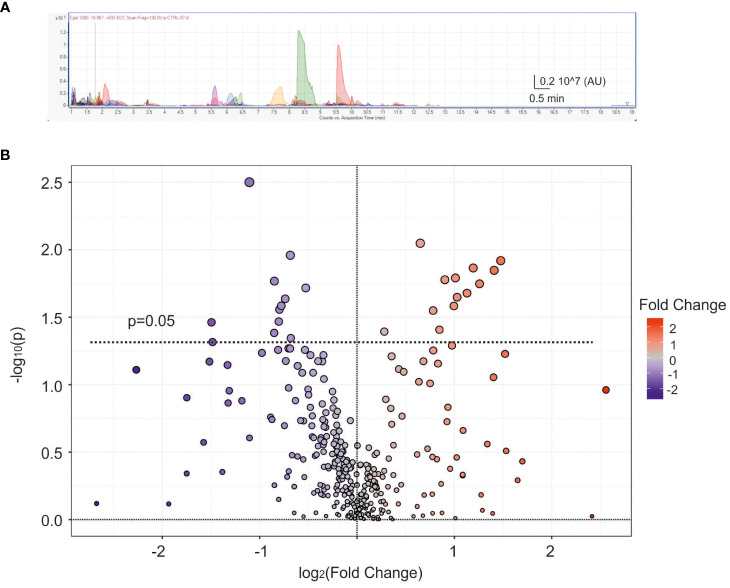
Metabolic analysis of follicular fluid. **(A)** Exemplificative spectral peak collection of follicular fluid obtained from a control patient. Different colours represent different metabolites AU=arbitrary units. **(B)** Average scatter Volcano plot displaying all metabolites dispersed based on the fold change (FC) and p-value. The dashed line represents p=0.05, with blue and red dots indicating down-regulated and up-regulated metabolites, respectively.

With the aim to investigate the possible role of VIP neurotransmission dysregulation in PCOS, we performed an untargeted metabolomic analysis to profile the polar compounds present in the samples, typically associated with neurotransmission. Using liquid chromatography-quadrupole time-of-flight mass spectrometry (LC-QTOF MS) a total of 333 different metabolites (137 named biochemicals and 196 unnamed biochemicals) were screened. An exemplificative spectral peak collection is displayed in **Figure 3A**. The analysis performed in the whole pool (9 PCOS and 10 non-PCOS samples used in **Figures 1** and **2**) was displayed in the Volcano plot (**Figure 3B**), where the fold of change versus p-value is plotted. Among the total 333 metabolites, 25 compounds resulted statistically different according to t-test (p<0.05). Specifically, we found a significant reduction of several metabolites related to 1-lysophosphatidylcholines (p<0.05, see **Supplementary Material 2A-1**), which have already been reported as biomarkers for the prediction follicular development (42).

**Figure 4:**
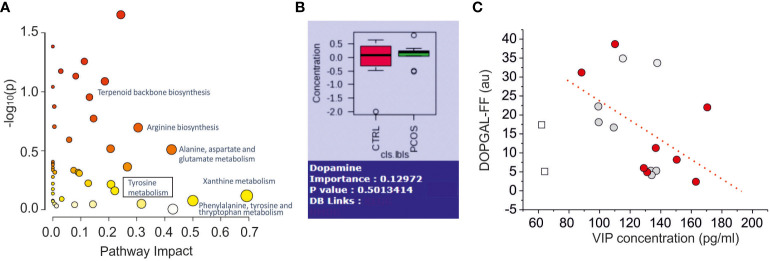
Metabolomics Pathway Analysis of Differential Metabolites in Follicular Fluid Between Normal and PCOS Patients. **(A)** Pathway analysis results of the 137 named metabolites. The square box indicates the pathway related to catecholaminergic metabolites (Tyrosine metabolites). **(B)** Comparation of dopamine concentration between control and PCOS group (no significance found p=0.50). **(C)** Scatter plot of noradrenergic metabolites (DOPGAL) versus VIP levels. The dashed line represents the linear correlation with the data. The empty square data points represent outlier data not included in linear regression. The grey and red dots indicate normal and PCOS patients, respectively.

By using Kegg and HMDB libraries, applied to identify the metabolites, we performed a pathway analysis. In **Figure 4A** we displayed the principal pathway differences scattered based on pathway impact (which accounts for the number of metabolites altered with respect to the overall metabolites pathway) versus the cumulative p-values. Among them, we noted an alteration of the tyrosine metabolism (box in **Figure 4A**), that is associated with catecholaminergic neurotransmission. Since VIP was found to regulate the levels of NA and its metabolites in the ovaries of the rat (18) we performed a correlation study between VIP levels and the identified catecholaminergic metabolites, in order to find a dysfunctional association in PCOS. Among the numerous compounds present in the pathway of tyrosine, we identified L-tyrosine, dopamine, 3,4-dihydroxyphenylglycolaldehyde (DOPGAL) and 3-methoxy-4-hydrohyphenylacetaldehyde (homovanilline), but no clear evidence of modification of the concentrations of these compounds in FF of PCOS and control patients was found. NA was not detected in our samples, probably because the concentration is below the limit of detection of our technology, experimentally established at (0.22μg/ml), as previously reported in literature (43). Similar results were obtained by using spectrometric analysis with LC-MS with a single quadrupole detector and NA was not detected again (see **Supplementary Figure 3**).

The other catecholaminergic neurotransmitter dopamine, in contrast, was detected and quantified (limit of detection: 0.1μg/mL), but no difference was found between the two groups (**Figure 4B**). Interestingly, we found a negative correlation between VIP-FF and DOPGAL-FF concentrations (r=0.47, dashed line in **Figure 4C**), according to the effect of VIP antagonist effect on NA metabolism in the ovary of the rat (18).

## Discussion

The main results herein reported indicate an increased VIP concentration in FF of PCOS patients compared to non-PCOS patients which underwent IVF procedure. However, even if the VIP presence in FF has been already reported (44), the difference in the concentration amoung in PCOS patients was first reported in this paper. This difference is in accordance with neuroanatomical studies that found increased VIP-ergic fibers in the ovarian stroma of PCOS patients (9).

VIP acts as a neurotransmitter in the autonomic nervous system of the ovary in both in sympathetic and in parasympathetic fibers, but also in sensory neurons (45, 46). VIP-ergic fibers are present in ovarian follicles at the level of the theca cells and in all stages of development where they also play a role in the steroidogenesis regulation of reproductive function (10). In the follicular cells VIP activates cAMP/PKA Type I pathway and it increases the expression and function of STeroidogenic Acute Regulatory (STAR) protein (47) and the production of P4 and E2 (28, 44). In PCOS patients, the signaling associated with P4 is altered, as a consequence of an increased expression of progesterone receptor (PGR) and pentraxin 3 (PTX3) (48). VIP stimulates DNA synthesis and estradiol secretion in cultured human granulosa/luteal cells (44). Plasma androgen concentration was quickly increased after VIP intravenous perfusion in healthy woman (49). The effect of VIP on androgens could be linked to an hyperandrogenic phenotype found in PCOS patients (50). Furthermore VIP-increased cAMP levels in granulosa cells contributes to the differentiation process in the acquisition of FSH responsiveness (51).

Specifically, a subgroup of women affected by PCOS showed an alteration of several physiological parameters regulated by the sympathetic nervous system. Indeed, among 50% of women with PCOS a higher resting MSNA (muscle sympathetic nerve activity) was found (52, 53). Similar result was found by Lambert et al. together with endothelial dysfunction (54).

Our results show that the increased AMH is directly proportional to the increase in VIP concentration. AMH participates in two crucial phases of follicles development that are the inhibition of the recruitment of primordial follicles into the pool of growing follicles and the decrease of the responsiveness of growing follicles to FSH (55, 56). The ability of VIP to modulate FSH responsiveness could explain the lack of correlation between the rFSH starting dose and AMH levels when algorithm were applied to predict in IVF (55). It will be interesting to verify the possibility of developing a new algorithm accounting also for plasma VIP levels.

We further elucidated if the E2 level could be correlated to the VIP elevation dected. Our results showed that FF-E2 concentration was higher in PCOS women, in accordance with previously reported findings (57, 58), but no correlation was found with VIP, suggesting the scantly role of VIP in E2 production, while further studies are needed to understand the possible role in other steroidogenic pathways, such as androgens. In contrast, FF-E2 is correlated with E2 concentration in the plasma. This latter aspect is not to be surprised, since FF composition derives both from serum diffusion and metabolites produced from the follicular cells and from the metabolic reactions that occur inside of it. The correlation between plasma and follicular concentration can be observed for some compounds, probably on the base of the lipophilic profile of the investigated compound (as already reported for example for vitamin D), but the exact relationship between abundance in FF and chemical physical properties requires further investigation.

Another important result obtained by performing a metabolomic analysis was the reduced presence of noradrenergic metabolite DOPGAL that is inversely correlated with VIP.

In this context, LC-MS/MS is a powerful but challenging tool to perform an untargeted metabolomic analysis, with the limitation of lack of reference libraries useful for compound identification (59). Recently the metabolomic analysis of FF reported an alteration in lysophosphatidylcholines in the presence of small follicles, as we also observed in PCOS patients (**Supplementary Figure 3**). The reduced concentration of DOPGAL in PCOS patients.

**Figure 5:**
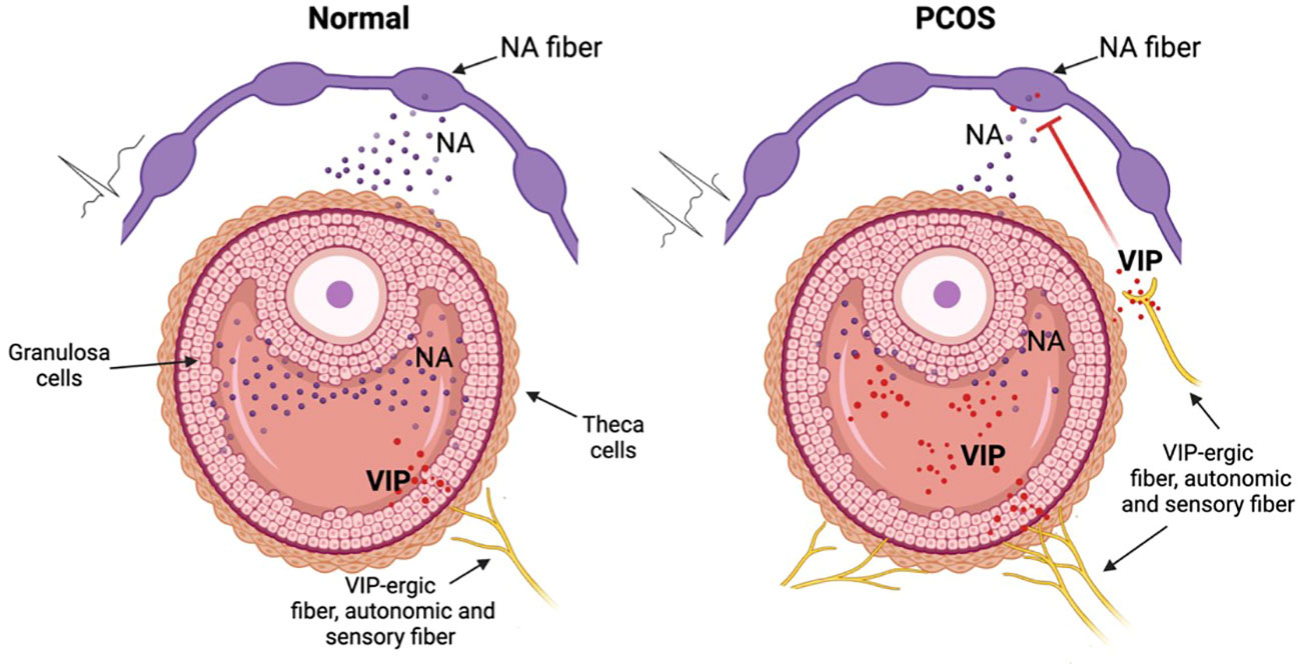
Hypothetic mechanism of VIP dysregulation in the PCOS disorder. In normal conditions (left picture) the NA sympathetic fibers release NA that is involved in correct folliculogenesis. In the PCOS condition (right picture) VIP, derived from autonomic (sympathetic and parasympathetic) or from sensory neurons, inhibits NA release thus modifing the normal folliculogenesis. This inhibition nullifies the increased excitability originated from the increase of density and of eccitability of sympathetic system in the PCOS ovary. Figure created with BioRender.com.

The reduced concentration of DOPGAL in PCOS patients displays a downregulation of sympathetic function, in contrast to general idea that in PCOS there is a hyperactivity of sympathetic system. How can we explain this paradox? In the model, reported in **Figure 5**, we suggest that VIP could represent an inhibitor of NA tone, stimulating the presynaptic VIP receptors in NA fibers, in a similar way to α2 adrenoreceptor. In this contest, the presynaptic inhibition, promoted by VIP, decreases the ability of adrenergic synapsis to convert excitability in NA release. The overall sympathetic activity depends on the balance of excitability (that increases in PCOS pathology) and the presynaptic modulating/inhibiting tone (suggested by our results). Our opinion is that, although several parameters indicate an increase of sympathetic tone (increase of innervation density, electrophysiological firing, etc.) in some subgroups of PCOS women the VIP-ergic tone determines a hypo functionality of catecholaminergic synapsis. Other experiments are needed to demonstrate this hypothetical model of neuromodulation that may clarify the pathophysiological role of VIP in PCOS.

## Data availability statement

The raw data supporting the conclusions of this article will be made available by the authors, without undue reservation.

## Ethics statement

The studies involving humans were approved by the Local Ethic Commitee - CER Umbria Protocol number: 27139 and registered in the ClinicalTrialsgov. Protocol Registration System (identifier: NCT0595891). The studies were conducted in accordance with the local legislation and institutional requirements. The participants provided their written informed consent to participate in this study.

## Author contributions

BF: Conceptualization, Data curation, Funding acquisition, Supervision, Writing – original draft, Writing – review & editing. LS: Data curation, Investigation, Methodology, Writing – original draft. EG: Data curation, Investigation, Writing – review & editing. DF: Data curation, Formal analysis, Software, Validation, Writing – review & editing. PS: Methodology, Resources, Software, Validation, Writing – review & editing. RMP: Writing – review & editing, Methodology, Investigation, Software. HBRA: Writing – review & editing, Methodology, Investigation, Software. DB: Writing – review & editing, Methodology, Validation, Data Curation, Investigation. SG: Methodology, Project administration, Validation, Writing – review & editing. CA: Conceptualization, Resources, Writing – review & editing. EC: Data curation, Validation, Writing – review & editing. EC: Validation, Writing – review & editing. AM: Conceptualization, Resources, Supervision, Writing – original draft.

## Supplementary material

Supplementary Figure 1 VIP concentration estimated in FF sample during defrosting process. To be noted the significant reduction (p<0.05) observed in any sample that demonstrates VIP biological instability in.

Supplementary Figure 2 Principal dowregulated metabolites in PCOS versus non PCOS.

Supplementary Figure 3 Chromatogram of noradrenaline standard sample (upper) and follicular fluid (lower) by using single quadrupole detector. Note that NA was not detected in FF.
