# Supplementary material for: Increased Vasoactive Intestinal Peptide (VIP) in polycystic ovary syndrome patients undergoing IVF
Source: Front Endocrinol (Lausanne). 2024 May 7;15:1331282. doi: 10.3389/fendo.2024.1331282 (PMC11106456; doi:10.3389/fendo.2024.1331282)
Supplement: Supplementary Figure 1 — VIP concentration estimated in FF sample during defrosting process. To be noted the significant reduction (p<0.05) observed in any sample that demonstrates VIP biological instability in. [file DataSheet_1.docx]

**SUPPLEMENTARY MATERIAL**


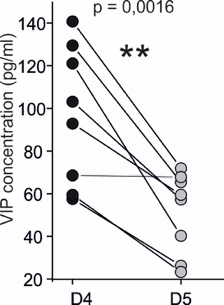


**Supplementary Figure 1. VIP concentration estimated in FF sample during defrosting process.** To be noted the significant reduction (p<0.05) observed in any sample that demonstrates VIP biological instability in


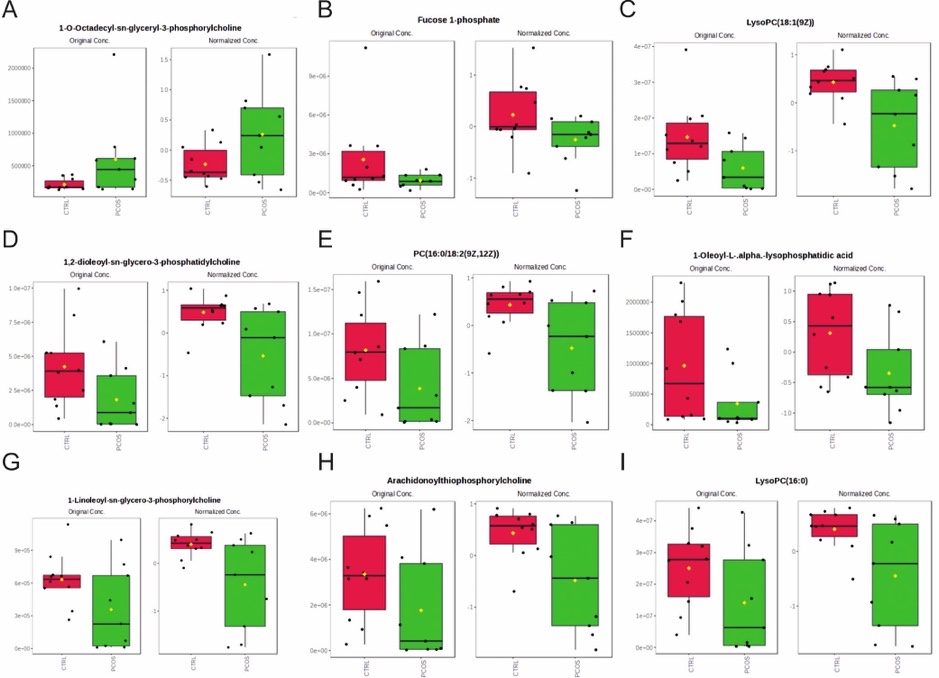


**Supplementary Figure 2. Principal downregulated metabolites in PCOS versus non PCOS.**

**
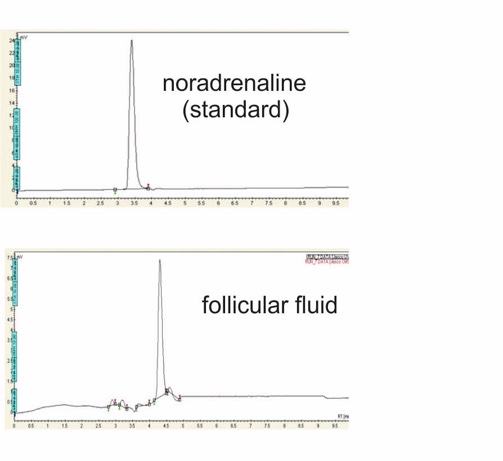
**

**Supplementary Figure 3.** **Chromatogram of noradrenaline** standard sample (upper) and follicular fluid (lower) by using single quadrupole detector. Note that NA was not detected in FF.
